# The involvement of C5a in the progression of experimental arthritis with *Porphyromonas gingivalis* infection in SKG mice

**DOI:** 10.1186/s13075-018-1744-3

**Published:** 2018-11-03

**Authors:** Syuichi Munenaga, Kazuhisa Ouhara, Yuta Hamamoto, Mikihito Kajiya, Katsuhiro Takeda, Satoshi Yamasaki, Toshihisa Kawai, Noriyoshi Mizuno, Tsuyoshi Fujita, Eiji Sugiyama, Hidemi Kurihara

**Affiliations:** 1 Department of Periodontal Medicine, Graduate School of Biomedical & Sciences, Hiroshima University, 1-2-3, Kasumi, Minami-ku, Hiroshima, 734-8553 Japan; 2 Division of Rheumatology, Kurume University Medical Center, 155-1 Kokubu-machi, Kurume, 839-0863 Japan; 3 Department of Periodontology, Nova Southeastern University College of Dental Medicine, 3200 South University Drive, Fort Lauderdale, FL 33328 USA; 4 Department of Clinical Immunology and Rheumatology, Hiroshima University Hospital, 1-2-3 Kasumi, Minami-ku, Hiroshima, 734-8553 Japan

**Keywords:** Arthritis, C5a, *Porphyromonas gingivalis*, SKG mice

## Abstract

**Background:**

Epidemiological evidence to suggest that periodontal disease (PD) is involved in the progression of rheumatoid arthritis (RA) is increasing. The complement system plays a critical role in immune responses. C5a has been implicated in chronic inflammatory diseases, including PD and RA. *Porphyromonas gingivalis* is the major causative bacteria of PD and can produce C5a. Therefore, it is hypothesized that *P. gingivalis* infection is involved in the progression of RA by elevating C5a levels. In the present study, *P. gingivalis*–infected RA model mice were established to investigate the involvement of C5a.

**Methods:**

SKG mice orally infected with *P. gingivalis* were immunized with intraperitoneal injection of laminarin (LA) to induce arthritis. Arthritis development was assessed by arthritis score (AS), bone destruction on the talus, histology, and serum markers of RA. In order to investigate the effects of serum C5a on bone destruction, osteoclast differentiation of bone marrow mononuclear cells was examined by using serum samples from each group of mice. The relationship between C5a levels and antibody titers to periodontal pathogens in patients with RA was investigated by enzyme-linked immunosorbent assay.

**Results:**

*P. gingivalis* oral infection increased AS, infiltration of inflammatory cells, bone destruction on the talus, and serum markers of RA in mice immunized with LA. The addition of serum from LA-injected mice with the *P. gingivalis* oral infection promoted osteoclast differentiation, and the addition of a neutralization antibody against C5a suppressed osteoclast differentiation. C5a levels of serum in RA patients with positive *P. gingivalis* antibody were elevated compared with those in RA patients with negative *P. gingivalis* antibody.

**Conclusions:**

These results suggest that *P. gingivalis* infection enhances the progression of RA via C5a.

## Background

Rheumatoid arthritis (RA) is a systemic autoimmune disease that leads to synovial inflammation, cartilage damage, and bone destruction [1, 2]. In recent years, the involvement of periodontal disease (PD) in the pathogenesis of RA has attracted attention. PD is characterized by chronic inflammation due to interactions between periodontopathogenic bacteria and host immune responses. Periodontopathogenic bacteria are composed of a group of Gram-negative anaerobic organisms. *Porphyromonas gingivalis*, *Treponema denticola*, and *Tannerella forsythia* play a central role in the pathogenesis of PD and these bacteria are the so-called “red complex” [3]. Previous studies evaluated the systemic effects of PD [4, 5].

RA and PD have immunologically common features. Both disease conditions involve inflammatory cytokines, T helper 17 (Th17) cells, and osteoclast-mediated bone destruction [6, 7]. They also show the overlap of environment and genetic factors, such as major histocompatibility complex (MHC) class II HLA-DRB1 epitopes and smoking, respectively [8]. Although epidemiological evidence to support the link between PD and RA is increasing, the underlying mechanisms remain unclear [9–11]. These two diseases are prevalent inflammatory diseases, and many patients exhibit chronic inflammation, the loss of function, and disability in daily life. Therefore, the involvement of PD in the pathogenesis of RA needs to be clarified in more detail and may lead to the development of future preventive and therapeutic strategies.

Complement plays an important role in host defenses and inflammation by affecting innate and adaptive immune cells [12]. The involvement of the complement system in the pathogenesis of both diseases seems to be important. There are three pathways of complement activation: the classic pathway, lectin pathway, and alternative pathway. All three pathways result in the generation of C3 convertase, which leads to the activation of effector molecules such as C3a and C5a. C5a mediates the recruitment and activation of myeloid cells such as neutrophils, monocytes, and macrophages via C5aR. Among complement factors, C5a is the most powerful effector molecule and has been implicated in chronic inflammatory diseases, including PD and RA [13, 14]. A large number of studies support the C5a–C5aR axis being a critical factor for both diseases.

In patients with RA, the amount of C5a and number of C5aR-positive cells were found to be increased in synovial tissues [15, 16]. C5aR-deficient mice and C5aR antagonist (C5aRA; PMX53)-treated mice in a collagen-induced arthritis (CIA) model, which is a classic model of inflammatory arthritis, were shown to be resistant to synovial inflammation and bone destruction in joints [17, 18]. In PD, when mouse models of *P. gingivalis*–induced periodontitis were locally treated with a C5aRA, gingival inflammation and alveolar bone destruction were significantly less severe than in the same model without C5aRA [19, 20]. *P. gingivalis* generates biologically active C5a by its Arg-specific gingipains that have C5 convertase-like activity [21]. Therefore, *P. gingivalis* can interfere with host immunity, leading to the development of PD. Furthermore, C5a is known to be involved in bone immunopathology. C5a may induce osteoclast differentiation from blood mononuclear cells, and C5aR is required for osteoclast differentiation [22, 23]. Hence, C5a may function as a key factor in the potential link between the two diseases and have important implications for therapeutic approaches against them. As such, the involvement of C5a with *P. gingivalis* infection in the progression of RA needs to be clarified.

SKG mice, in which a point mutation of the gene encoding ZAP-70 exists on the BALB/c background, develop an arthritis whose features closely resemble those of human RA [24]. Our group previously reported that arthritis was exacerbated in SKG mice infected with *P. gingivalis* via intraperitoneal (i.p.) injection. One of the underlying mechanisms was the promotion of osteoclast differentiation [25]. Arthritis in SKG mice was induced by an injection of β-glucan, such as laminarin (LA) [26]. β-glucan activates innate immunity and the complement pathway, leading to the generation of C5a. C5a signaling induces the development of Th17 cells, which play a critical role in the development of arthritis through the induction of cytokines such as interleukin-1beta (IL-1β), IL-6, and granulocyte-macrophage colony-stimulating factor (GM-CSF) in macrophages [27]. C5aR-deficient SKG mice inhibit arthritis and Th17 cell development. Therefore, the C5a–C5aR axis plays an important role in regulating Th17-mediated autoimmune arthritis.

In the present study, we hypothesized that the C5a elevation in *P. gingivalis* infection is involved in the progression of RA. To test this hypothesis, SKG mice with *P. gingivalis* oral infection were established and investigated whether *P. gingivalis* oral infection affects the development of experimental arthritis via elevations in C5a levels. In addition, the association between antibody titers to *P. gingivalis* and C5a levels in patients with RA was examined.

## Methods

### Preparation of bacteria

*P. gingivalis* W83 was purchased from the American Type Culture Collection (Manassas, VA, USA). *P. gingivalis* W83 was cultured on a sheep blood agar plate at 37 °C using the Anaeropack system (Mitsubishi Gas Chemical, Tokyo, Japan). After a 2-day incubation, *P. gingivalis* W83 was inoculated in 40 mL of trypticase soy broth supplemented with 1% yeast extract, hemin (200 μg), and menadione (20 μg). Bacteria were harvested in the exponential growth phase and washed with phosphate-buffered saline (PBS).

### Induction of arthritis and periodontitis

Female 6- to 8-week-old SKG mice (Clea Japan, Inc., Tokyo, Japan) were immunized by an i.p. injection of LA (Sigma-Aldrich, St. Louis, MO, USA, 10 mg/100 μL/mouse) to induce arthritis. Periodontitis was also induced in SKG mice by an oral inoculation of 10^8^ colony-forming units *P. gingivalis* W83. *P. gingivalis* W83 was suspended in 50 μL of PBS containing 2% carboxymethylcellulose (CMC) and inoculated repeatedly every 3 days for 42 days. This term was set in accordance with a previous bone resorption mouse model infected by *P. gingivalis* [19]. The number of administered bacteria was determined by considering body weight and the number of bacteria in the saliva of patients with periodontitis. Mice were divided into four groups (Ctrl group: PBS administration; Pg group: Pg inoculation; LA group: LA injection; Pg/LA group: Pg inoculation + LA injection). Animal experiments were approved by the ethics committee of Hiroshima University (approval A12–15). All experiments were performed three times (*n* = 6 per group).

### Assessment of periodontitis

In order to measure alveolar bone loss (ABL), maxilla halves were assessed as described previously [28].

### Measurement of antibody titers to periodontopathogenic bacteria

Antibody titers to periodontopathogenic bacteria (*P. gingivalis*, *Prevotella intermedia*, *Treponema denticola*, *Tannerella forsythia*, and *Aggregatibcter actinomycetemcomitans*) were measured by enzyme-linked immunosorbent assay (ELISA). The antigens of the fraction of outer membrane proteins from periodontopathogenic bacteria were coated onto 96-well Maxisorp Nunc Immunoplates (Nunc, Roskilde, Denmark) in sodium bicarbonate buffer, pH 9.4, overnight at room temperature (RT). After blocking each well with 1% bovine serum albumin (BSA) in PBS supplemented with 0.05% Tween 20 (PBST) at RT for 1 h, human serum (3200-fold dilution) or mouse serum (100-fold dilution) were applied to each well at RT for 2 h. Wells were washed three times by PBST and incubated with a human or mouse horseradish peroxidase (HRP)-conjugated secondary antibody (2000-fold dilution in PBST) at RT for 1 h. After the final washes, citrate-phosphate buffer, pH 5.0, containing 0.3% hydrogen peroxide and 0.25% o-phenylenediamine was added. The coloring reaction was allowed to continue for 15 min and was stopped by the addition of 25 μL of 2 N sulfuric acid. Absorbance at 405 nm was measured by using a plate reader (Bio-Rad Laboratories, Hercules, CA, USA).

### Clinical assessment of experimental arthritis

Arthritis score (AS) was monitored weekly and recorded using a previously published system [24]: 0, no swelling or redness; 0.1, swelling or redness of the digits; 0.5, mild swelling or redness (or both) of the wrist or ankle joints; 1, severe swelling of the large joints. Scores of the affected joints were totaled for each mouse; the maximum total score per mouse was 6.0, and the minimum was 0.

### Histological examination

Ankle joints were isolated at the end of the experiment in 4% paraformaldehyde for 24 h, decalcified in 10% ethylene diamine tetrameric acid (EDTA) for 42 days, and embedded in paraffin. Seven-micrometer-thick tissue sections were stained with hematoxylin and eosin (HE), Safranin O, and a tartrate-resistant acid phosphatase (TRAP) staining kit (Takara Bio, Inc., Shiga, Japan). The severities of inflammation and cartilage damage were scored using published criteria [29]. The number of TRAP-positive cells in a randomly selected diseased site (three sites per picture) was counted and compared among the different groups.

### Micro-computed tomography analysis

In the micro-computed tomography (micro-CT) analysis, mice were scanned by using a Skyscan 1076 (Bruker, Kontich, Belgium) to evaluate bone destruction. Joint samples were scanned and reconstructed at 18 μm^3^ voxels by using a micro-CT system.

### Measurement of the factors in serum by ELISA

ELISAs for human C5a (#DY2037; R&D, Minneapolis, MN, USA), mouse C5a (#EK0987; Boster, Pleasanton, CA, USA), mouse IL-6 (#431304; BioLegend Inc., San Diego, CA, USA), mouse matrix metalloproteinase-3 (MMP-3, #MMP300; R&D), and mouse anti-citrullinated protein antibody (ACPA) (#ORG601 Orgentec, Chicago, IL, USA) in serum were performed in accordance with the instructions of the manufacturers. The limits of detection for each analyte were as follows: human C5a, 31.3 pg/mL; mouse C5a, 15.6 pg/mL; mouse IL-6, 7.8 pg/mL; mouse MMP-3, 0.312 ng/mL; and mouse ACPA, 0 U/mL.

### Immunohistochemical analysis

Fixed tissues were embedded in paraffin wax, at a thickness of 7 μm, mounted on slides. Slides were incubated with a primary goat polyclonal antibody for C5a (#sc-21,944; Santa Cruz Biotechnology, Inc., Dallas, TX, USA) at 4 °C overnight. Slides were then washed three times in PBST and incubated with a goat HRP-conjugated secondary antibody (1:200) at RT for 1 h. After three washings, color development was achieved with 3,3′-diaminobenzidine. Photographs were taken with a Nikon Eclipse E600 (Nikon, Tokyo, Japan).

### Collection of bone marrow mononuclear cells

Bone marrow mononuclear cells (BMCs) from the femurs of SKG mice were isolated by density gradient centrifugation with Histopaque-1083 (Sigma-Aldrich) in complete Dulbecco’s modified Eagle’s medium (DMEM) containing 10% fetal bovine serum (FBS) (Invitrogen, Carlsbad, CA, USA), antibiotics (penicillin, streptomycin, and gentamicin; Invitrogen), and L-glutamine.

### Quantitative RT-PCR

Total RNA was extracted from the liver and BMCs by using RNAiso (Takara Bio, Inc.) in accordance with the protocol of the manufacturer. Briefly, one microgram of total RNA was used for reverse transcription by ReverTra Ace^®^ for reverse transcription-polymerase chain reaction (RT-PCR) (cat. no. TRT-101, Toyobo, Osaka, Japan). Real-time PCR was performed by StepOne Plus (Applied Biosystems, Carlsbad, CA, USA). Amplification conditions were described previously [30]. Template cDNA was mixed with the Core Reagent Fast SYBR^®^ Master Mix system (Applied Biosystems), distilled water, and a primer (10 pmol). The following primer sets were used for real-time PCR: C5 forward, 5′-TTTCAGCACCCAAAATCCTC-3′ and C5 reverse, 5′-CGCGTTTTGGAATTTGTTTT-3′; tumor necrosis factor (TNF) receptor-associated factor 6 (TRAF6) forward, 5′-CTGCAAAGCCTGCATCAT-3′ and TRAF6 reverse, 5′-AATGTGTGTATTAACCTGGC-3′; nuclear factor of activated T cells, cytoplasmic 1 (NFATc1) forward, 5′-TCATCCTGTCCAACACCAAA-3′ and NFATc1 reverse, 5′-TTGCGGAAAGGTGGTATCTC-3′; MMP-9 forward, 5′-CTGGACAGCCAGACACTAAAG-3′ and MMP-9 reverse, 5′-CTCGCGGCAAGTCTTCAGAG-3′; 18 s rRNA forward, 5′-GTAACCCGTTGAACCCCATT-3′ and 18 s rRNA reverse, 5′-CCATCCAATCGGTAGTAGCG-3′. Relative expression levels were calculated by standard curve methods and ΔΔCt methods, and 18S ribosomal RNA was used as the internal control.

### Osteoclast differentiation and resorption assay

BMCs were seeded on 96-well plates for osteoclast differentiation assay or osteo assay plates for resorption assay at a density of 1.0 × 10^5^ cells per well and cultured in alpha-modified Eagle’s minimum essential medium (α-MEM) with 15% FBS containing 20 ng/mL M-CSF (#315–02; Peprotech, Rocky Hill, NJ, USA) and 50 ng/mL murine soluble recombinant receptor activation of nuclear factor kappa-B ligand (sRANKL, #315–11; Peprotech). After 2 days, BMCs were cultured in the presence of 20 ng/mL M-CSF and 50 ng/mL sRANKL with 10% FBS and 5% mouse serum for 3 days. Sera were prepared from each group of mice. In regard to the neutralization of C5a, 0.5 μg/mL anti-mouse C5a antibody (#MAB21501; R&D) or rat IgG2 isotype control (#MAB006; R&D) was added to the culture medium. After 3 days, differentiated osteoclasts were identified by TRAP staining and TRAP-positive multinucleated (more than three nuclei) cell numbers were evaluated. Resorption areas were measured by ImageJ after removal of the cells with 1 M NH_4_OH.

### Participants

Sera were collected from 40 patients at the Department of Clinical Immunology and Rheumatology, Hiroshima University Hospital. All of the patients with RA fulfilled the 2010 American College of Rheumatology criteria for RA.

### Ethics approval

All patients provided written informed consent prior to enrollment. This study was approved by the ethics committee of Hiroshima University Hospital (#1017).

### Evaluation of antibody titers of patients by ELISA units

The antibody titers to periodontal pathogens in human were expressed as ELISA units. ELISA units were measured as described previously [31]. A positive antibody response was defined as a standard deviation of more than 2 above the mean ELISA units of the age-similar healthy hospital personnel.

### Statistical analysis

Data are expressed as the mean ± standard error of the mean. Statistical analyses between two groups were performed by using the Mann–Whitney *U* test in the case of a non-normal distribution. For multiple comparisons, the Tukey-Kramer test or Bonferroni-corrected Mann–Whitney *U* test was used. Pearson’s or Spearman’s correlation coefficient was calculated for the correlations performed. In all tests, a *P* value of less than 0.05 was considered significant.

## Results

### Periodontitis induced by *P. gingivalis* oral infection increases the severity of experimental arthritis

**Fig. 1:**
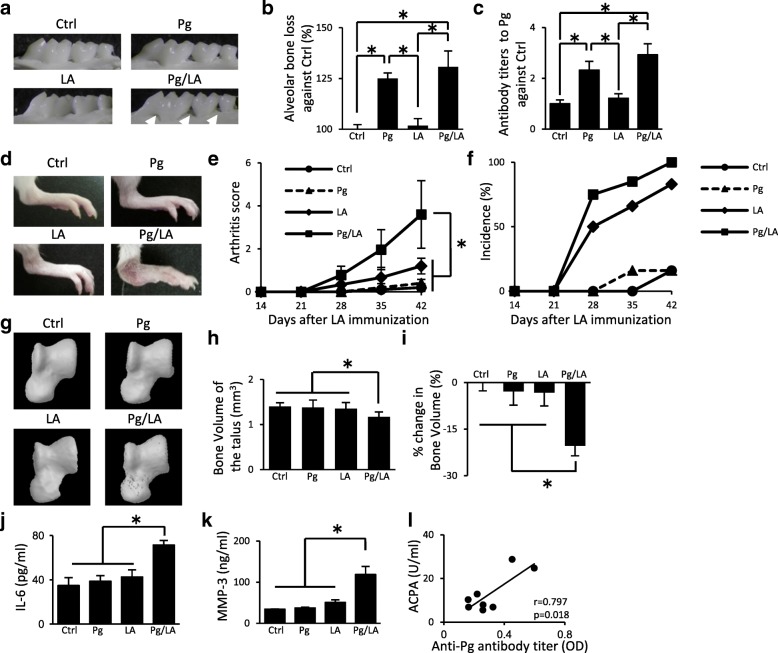
SKG mice received an oral inoculation of *Porphyromonas gingivalis* (Pg) (10^8^ colony-forming units per mouse) repeatedly every 3 days for 42 days after the laminarin (LA) immunization: control (Ctrl) *n* = 6, Pg n = 6, LA n = 6, Pg/LA *n* = 8. **a** Representative images of the palatal surfaces of the maxillary molars from the four groups (arrows indicate the resorption site). **b** Alveolar bone loss was analyzed by measuring the distance from the cemento-enamel junction to the alveolar bone crest on palatal surfaces. **c** Antibody titers to *P. gingivalis* in serum were measured by enzyme-linked immunosorbent assay. **d** Representative figures of hind paws for the four groups showing joint inflammation. **e, f** The severity and incidence of arthritis were scored by using Sakaguchi’s arthritis score. **g** Representative micro-computed tomography images of the talus. **h, i** Bone volume of the talus and percentage changes in bone volume relative to Ctrl mice. **j, k** Serum concentrations of interleukin-6 (IL-6) and matrix metalloproteinase-3 (MMP-3) in the four groups. **l** The correlation between the serum level of anti-citrullinated protein antibody (ACPA) in the Pg/LA group and anti-Pg antibody titer. Data represent the mean ± standard deviation (**b, c**, and **d**) or mean ± standard error of the mean (**a**, and **e–k**) of 6–8 mice per group. Statistical analyses were performed by using the Tukey-Kramer test, Bonferroni-corrected Mann–Whitney *U* test, or Pearson’s correlation coefficient (*P <0.05). Abbreviation: *OD* optical density

In order to evaluate the induction of periodontitis, ABL and antibody titers to *P. gingivalis* in SKG mice after the oral infection of *P. gingivalis* were analyzed. The severity of ABL was remarkably greater in the Pg and Pg/LA groups than in the Ctrl and LA groups (Pg group: 125 ± 2.68% increase, Pg/LA group: 130.7 ± 7.86% increase against Ctrl, respectively) (Fig. 1a, b). The inoculation of the Pg and Pg/LA groups resulted in an increase in antibody titers to *P. gingivalis* in serum (Pg group: 2.32 ± 0.34-fold increase, Pg/LA group: 2.92 ± 0.43-fold increase against the Ctrl group, respectively) (Fig. 1c). However, no significant differences were observed between the Pg and Pg/LA groups. These results indicate that *P. gingivalis* oral infection induces periodontitis in SKG mice.

In order to examine the pathophysiological role of *P. gingivalis* oral infection in experimental arthritis, arthritis development was assessed in each group. The AS and incidence of arthritis were higher in the Pg/LA group than in the other groups in a time-dependent manner (Fig. 1d–f) (Ctrl group: 0.13 ± 0.07; Pg group: 0.15 ± 0.07; LA group: 1.2 ± 0.14; Pg/LA group: 3.6 ± 0.64). In order to investigate the influence of *P. gingivalis* oral infection on bone loss in joints, the bone properties of the talus as a parameter of focal bone loss were analyzed. Bone erosion of the talus in the Pg/LA group was observed in the micro-CT analysis (Fig. 1g). Talar bone volume was lower in the Pg/LA group than in the other groups (Ctrl group: 1.4 ± 0.08 mm^3^; Pg group: 1.36 ± 0.13 mm^3^; LA group: 1.35 ± 0.13 mm^3^; Pg/LA group: 1.11 ± 0.09 mm^3^), and the percentage of reduction in bone volume relative to the Ctrl group was the highest in the Pg/LA group (20.4 ± 3.19%) (Fig. 1h, i). Although the ankle joint was inflamed in the LA group, bone erosion, which was assessed using a micro-CT analysis, was not observed. Furthermore, increase of IL-6 and MMP-3 production in serum was greater in the Pg/LA group than in the other groups (IL-6: 54.3 ± 16.6 pg/mL; MMP-3: 118.6 ± 19.6 ng/mL) (Fig. 1j, k). The correlation between the production of ACPA in serum of Pg/LA group and anti-Pg antibody titer was analyzed. A positive correlation was observed (*r* = 0.797, *P* = 0.018) (Fig. 1l). These results suggest that *P. gingivalis* oral infection exacerbates experimental arthritis and bone destruction in SKG mice.

### Impact of *P. gingivalis* oral infection on the histopathology of arthritis

**Fig. 2:**
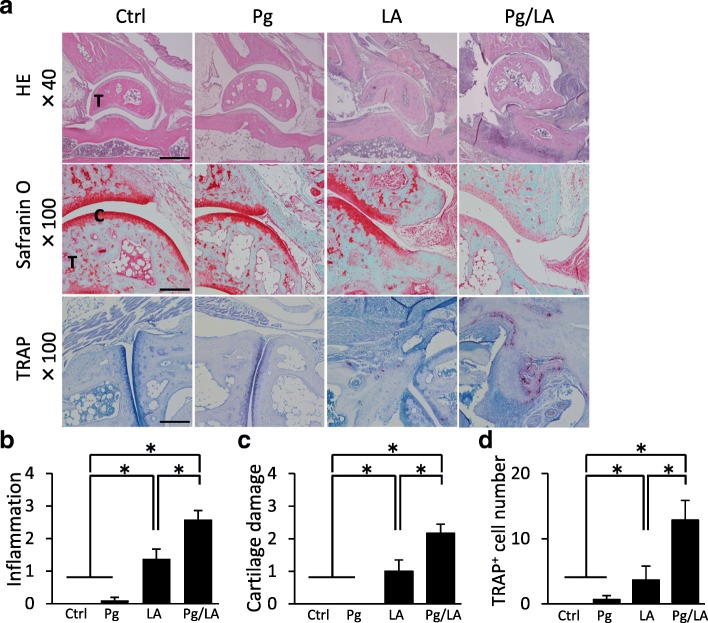
Histological assessment of ankle joints on day 42 from each group of mice. **a** Representative sections of the ankle joint stained with hematoxylin and eosin (HE), Safranin O, and tartrate-resistant acid phosphatase (TRAP). **b–d** Histological scores of inflammation, cartilage damage, and TRAP-positive cell numbers from each group of mice (n = 6 per group). Data represent the mean ± standard error of the mean. Statistical analyses were performed by the Bonferroni-corrected Mann–Whitney *U* test (*P <0.05). Original magnifications: 40×; scale bar = 500 μm, 100×; scale bar = 250 μm. Abbreviations: *C* cartilage, *Ctrl* control, *LA* laminarin, *Pg Porphyromonas gingivalis*, *T* talus

Histopathological changes in the ankle joint by HE staining, Safranin O staining, and TRAP staining were not observed in the Ctrl or Pg group (Fig. 2a). Inflammation, cartilage damage, and bone destruction were more severe in the Pg/LA group than in the other groups (Fig. 2a). These evaluation points were quantified by using a histological scoring system [29]. Inflammation, cartilage damage, and bone destruction in the ankle joint were more severe in the Pg/LA group than in the other groups (inflammation score: 2.67 ± 0.45; cartilage damage score: 2.17 ± 0.28; TRAP-positive cell number: 13.8 ± 8.74) (Fig. 2b–d). These results suggest that *P. gingivalis* oral infection in SKG mice promotes arthritis and leads to the bone destruction phase.

### Production of C5a in the serum and ankle joint

**Fig. 3:**
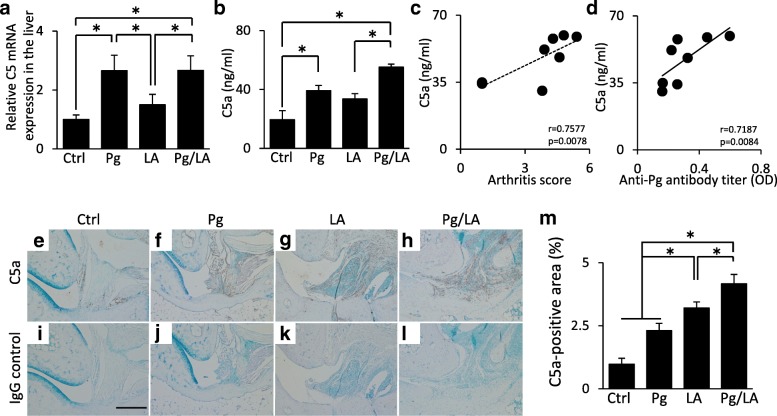
Serum, liver, and joints were collected from each group of mice at the end point of the experiment. **a** The mRNA expression of C5 in the liver was measured by quantitative reverse transcription-polymerase chain reaction. **b** Serum concentrations of C5a were measured by enzyme-linked immunosorbent assay. **c**, **d** Relationship between C5a levels and arthritis score or anti–*Porphyromonas gingivalis* antibody titers in the Pg/LA group. **e–l** Representative immunohistochemical images of C5a in the ankle joint. **m** The C5a-positive area was measured by ImageJ (n = 6 per group). Data represent the mean ± standard error of the mean. Statistical analyses were performed by Tukey-Kramer test, Pearson’s correlation coefficient (**d**), and Spearman’s correlation coefficient (**c**) (*P <0.05). Abbreviations: *Ctrl* control, *LA* laminarin, *OD* optical density, *Pg Porphyromonas gingivalis*

Previous studies indicated that C5a plays an important role in the progression of RA [17, 18]. Quantitative RT-PCR revealed that the mRNA expression levels of C5 in the liver were increased by *P. gingivalis* infection (Pg group: 2.66 ± 0.53-fold increase; Pg/LA group: 2.66 ± 0.49-fold increase against the Ctrl group, respectively) (Fig. 3a). C5a levels in serum were higher in the Pg and LA groups than in the Ctrl group. The increase of C5a levels observed in the Pg/LA group was prominent (Ctrl group: 19.4 ± 6.1 ng/mL; Pg group: 39.2 ± 3.54 ng/mL; LA group: 33.6 ± 3.57 ng/mL; Pg/LA group: 55.2 ± 1.99 ng/mL) (Fig. 3b). In the Pg/LA group, C5a levels in serum showed a positive correlation with AS and anti–*P. gingivalis* antibody titers (Fig. 3c, d).

Immunohistochemical-based quantification indicated that the positive area of C5a in the Pg/LA group was significantly increased (4.16 ± 0.23%) (Fig. 3e–m). The localization of C5a was close to the area of bone destruction in the punnus (Fig. 3h). These results support C5a being involved in bone destruction in inflamed joints in the Pg/LA group.

### Effects of C5a derived from serum on osteoclast differentiation

**Fig. 4:**
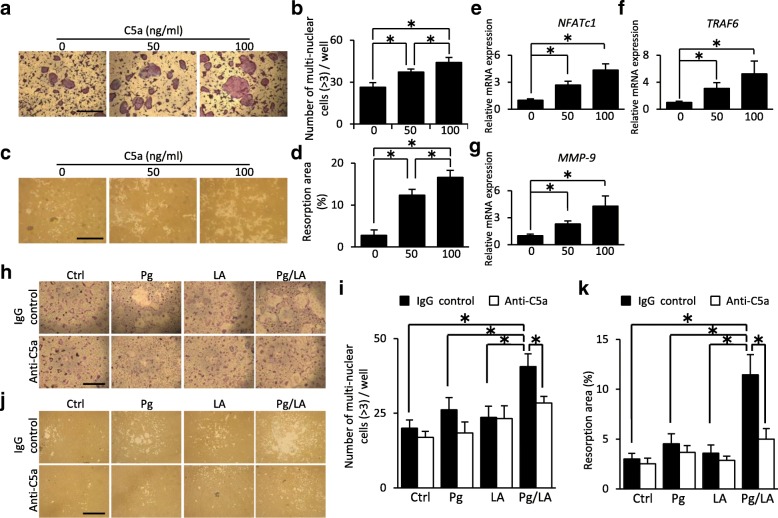
Bone marrow mononuclear cells (BMCs) were cultured in the presence of macrophage colony-stimulating factor and soluble recombinant receptor activation of nuclear factor kappa-B ligand to promote osteoclast differentiation. **a** Representative images of osteoclast differentiation with recombinant C5a (rC5a) by tartrate-resistant acid phosphatase staining. **b** The number of multi-nuclear osteoclast in each well (*n* = 10). **c** Representative images of bone resorption in the presence of rC5a by pit formation assay. **d** Bone resorption area by multi-nuclear osteoclast in each well was measured by ImageJ (*n* = 5). **e–g** Quantitative reverse transcription-polymerase chain reaction analysis of *NFATc1*, *TRAF6*, and *MMP-9* (*n* = 8). **h** Representative images of osteoclast differentiation of BMCs by the effect of serum derived from each group of mice in the presence of an isotype control antibody or anti-C5a antibody. **i** The number of multi-nuclear osteoclast in each well with or without serum derived from each group of mice (n = 10). **j** Representative images of bone resorption by the effect of serum derived from each group of mice in the presence of an isotype control antibody or anti-C5a antibody. **k** Bone resorption area by multi-nuclear osteoclast in each well was measured by ImageJ (n = 5). Statistical analyses were performed by the Student’s unpaired *t* test or Tukey-Kramer test (*P <0.05). Data represent the mean ± standard error of the mean. Original magnification: 40×; scale bar = 1000 μm

In order to investigate the contribution of C5a to bone destruction, osteoclast differentiation was examined by using BMCs in the presence of C5a. C5a could induce TRAP-positive cells in a dose-dependent manner (Fig. 4a, b). It was confirmed by pit formation assay that these cells have the function of bone resorption (Fig. 4c, d). C5a induced the mRNA expression of *NFATc1*, which is a master regulator of osteoclastogenesis, in a dose-dependent manner (Fig. 4e). In addition, the mRNA expression of *TRAF6* and *MMP-9*, which are osteoclast-associated markers, were induced (Fig. 4f, g). These results suggest that C5a can induce functional osteoclasts through the elevation of osteoclast-associated transcription factors, including *NFATc1*.

Furthermore, the addition of serum collected from the Pg/LA group induced functional osteoclasts more effectively than that from the other groups. These effects were blocked by the neutralizing antibody against C5a (Fig. 4h–k), indicating that the high level of C5a in the Pg/LA group was responsible for osteoclast differentiation. These results suggest that elevated C5a levels of serum in the Pg/LA group activate osteoclast differentiation.

### Relationship between C5a levels and antibody titers to periodontal pathogens in patients with RA

The demographics of RA participants in the present study are summarized in Table 1.

**Table 1:** Clinical conditions of participants (n = 40)

Parameters	Mean ± standard error
Age, years	60.6 ± 1.8
Female, percentage	65.0
CRP, mg/dL	0.57 ± 0.14
ESR, mm/hr	27.0 ± 3.51
VAS score, mm	40.5 ± 4.9
RF levels, IU/ml	73.9 ± 13.9
ACPA, U/ml	115.0 ± 35.3

Abbreviations: *ACPA* anti-citrullinated protein antibody, *CRP* C-reactive protein, *ESR* erythrocyte sedimentation rate, *RF* rheumatoid factor, *VAS* Visual Analogue Scale

**Fig. 5:**
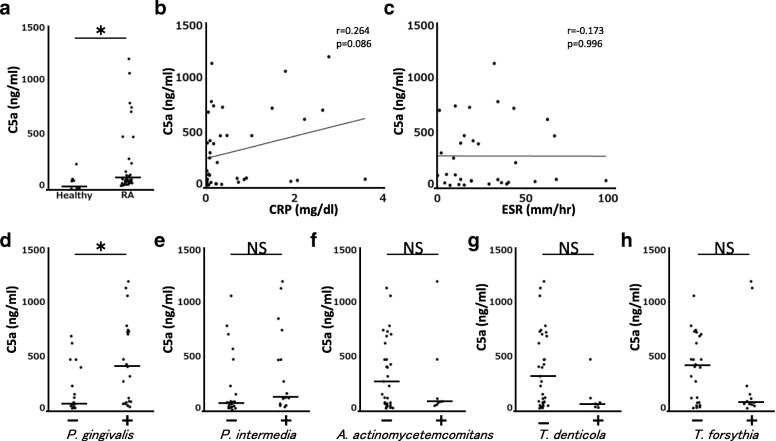
**a** C5a levels in serum from patients with rheumatoid arthritis (RA) and healthy control patients were measured by enzyme-linked immunosorbent assay (ELISA) (healthy, n = 8; patients with RA, n = 40). **b, c** Relationship between C5a levels and C-reactive protein (CRP) or erythrocyte sedimentation rate (ESR) in the serum from patients with RA. **d–h** C5a levels and antibody titers to periodontal pathogens (*Porphyromonas gingivalis* W83, *Prevotella intermedia*, *Treponema denticola*, *Tannerella forsythia*, and *Aggregatibactor actinomyvetemcomitance*) in serum from patients with RA were measured by ELISA. Bars show median of the data. Statistical analyses were performed by using Mann–Whitney *U* test and Pearson’s correlation coefficient (*P <0.05). Abbreviation: *NS* not significant

Patients with RA had significantly greater mean C5a levels in serum than healthy control participants (Fig. 5a). There was no significant correlation between C5a levels and C-reactive protein (CRP) or erythrocyte sedimentation rate (ESR) in serum (Fig. 5b, c). Thus, it was suggested that C5a was not solely dependent on inflammation in patients with RA. The relationship between C5a levels and antibody titers to periodontal pathogens in patients with RA was investigated. Antibody titers to periodontal pathogens differed among patients. RA patients with positive *P. gingivalis* antibody responses had significantly elevated C5a levels in serum compared with negative *P. gingivalis* antibody (Fig. 5d). The higher tendency of the levels of C5a in serum from the group of RA patients with negative *Treponema denticola*, *Tannerella forsythia*, and *Aggregatibcter actinomycetemcomitans* antibody was observed compared with serum from the group of positive *Treponema denticola*, *Tannerella forsythia*, and *Aggregatibcter actinomycetemcomitans* antibody patients. However, there was no statistical difference between positive and negative groups (Fig. 5e–h). These results suggest that *P. gingivalis* infection increases in C5a levels in patients with RA.

## Discussion

Epidemiological evidence to suggest that PD is involved in RA is increasing [32, 33]. Patients with RA show a significantly higher antibody response to *P. gingivalis* in comparison with systemically healthy individuals [34]. Serum levels of antibodies to *P. gingivalis* reflect the clinical and laboratory profiles of RA [35]. In addition, periodontal therapy was suggested to reduce the severity of RA as well as serum levels of antibodies to *P. gingivalis* [36]. Therefore, chronic *P. gingivalis* infection may influence the development of arthritis. *P. gingivalis* is the only human pathogen producing the peptidyl-arginine deiminase (PgPAD). Because of the PgPAD expression, *P. gingivalis* was considered as an important link between PD and RA [37]. However, the mechanisms responsible for this association remain unclear. *P. gingivalis* also has several virulence factors, such as fimbriae, lipopolysaccharide (LPS), and gingipain, which systemically activate immune response. *P. gingivalis* can also induce active C5a generation, which is one of the complement factors, via digestion by gingipain. Therefore, we focused on the involvement of complement in the progression of RA by *P. gingivalis* infection.

The complement system is activated by bacterial infection and inflammatory conditions, leading to the generation of anaphylatoxins such as C5a. Thus, C5a levels may be equally elevated in any of the periodontal pathogenic bacteria. However, our results showed the significantly elevated C5a levels of serum in RA patients with positive *P. gingivalis* antibody compared with that with negative *P. gingivalis* antibody. Moreover, no significant difference was observed between any of the other periodontal pathogens and C5a levels in serum (Fig. 5). Therefore, infection by *P. gingivalis* may have a greater potential to promote the generation of C5a than other periodontal pathogens in patients with RA. In order to account for this relationship, a large-scale clinical study is needed. It currently remains unclear how *P. gingivalis* infection is involved in the activation of C5a-mediated immune responses. Complement activation occurs through three different pathways (classic, lectin, and alternative) after bacterial infection and inflammatory responses as innate immunity [38]. It is possible that a proteinase derived from *P. gingivalis* (gingipain) or the immune complex consisting of bacterial antigens activates the complement cascade [39]. There is a report about the activation of C5a by gingipain [21]. The application of gingipain knockout mutant is a good way to clarify the involvement of C5a in the progression of Pg-induced RA. However, gingipain displays a diversity of effects on host cells, including the increase of attachment to gingival epithelial cells, hemin acquisition for growth, and proteolytical inactivation of cytokine [40]. Therefore, there is a possibility that the inhibitory effect of C5a cannot be observed by knockout mutant which is weak against host immune response.

The CIA model is the most widely accepted mouse model of RA and has contributed to clarify the pathology of RA. Previous reports indicated that *P. gingivalis* affects the immune system and the gut microbiota composition, leading to the arthritis development in the CIA model [41, 42]. Our group previously reported that *P. gingivalis* infection exacerbates arthritis in SKG mice via the promotion of osteoclast differentiation [25]. The cause of RA is systemic and abnormal immune responses [43]. SKG mice develop arthritis that closely resembles human RA, and arthritis in SKG mice is induced by an injection of β-glucan through the activation of systemic immune cells. Therefore, the use of SKG mice as an RA model has the advantage of inducing systemic immune responses. An i.p. injection of *P. gingivalis* to SKG mice was previously selected in an attempt to simplify the systemic effects of *P. gingivalis* in *in vivo* experiments [25]. However, orally applied bacteria are also known to influence bacterial flora in the digestive tract such as the mouth and gut. Since *P. gingivalis* is periodontopathogenic bacteria, an oral *P. gingivalis* inoculation model needs to be analyzed in order to physiologically investigate the effects of *P. gingivalis* infection.

In the present study, we established that arthritis was augmented by *P. gingivalis* oral infection in SKG mice. Severity of swelling, inflammation, cartilage damage, and bone destruction in joints was observed in the Pg/LA group (Figs. 1 and 2), and the expression of C5a in serum and joint was increased (Fig. 3). The increased expression of C5a by *P. gingivalis* oral infection is a potential cause of the acceleration of arthritis, particularly in the involvement of osteoclast differentiation. We confirmed that C5a can induce functional osteoclasts from BMCs and the mRNA expression of *NFATc1* in a dose-dependent manner. In addition, we demonstrated that the osteoclast differentiation of BMCs was potentiated more by serum from the Pg/LA group than from the other groups (Fig. 4). These results suggest that circulating C5a promoted osteoclast genesis, leading to bone destruction in joints. However, the biological activity of C5a is potently inhibited by removal of the carboxyl-terminal Arg, which is known as C5a des Arg [44]. Although C5a des Arg has many of the same functions, higher concentrations are needed in order to induce biological effects [45]. Moreover, other cytokines in serum, such as TNF-α, may participate in osteoclast differentiation [46, 47]. In order to validate the direct effects of C5a in serum, a neutralizing antibody against C5a was used in osteoclast differentiation. The osteoclast differentiation of BMCs, which were added to serum from the Pg/LA group, was significantly inhibited by the neutralizing antibody against C5a. Therefore, C5a in serum is involved in osteoclast differentiation. In addition to these results, BMCs that were harvested from arthritic SKG mice by an i.p. injection of *P. gingivalis* differentiated into markedly larger and multinuclear osteoclasts in our previous study [25]. There is a possibility that C5a in serum is involved in this mechanism.

In SKG mice, C5a drives Th17 cell differentiation and triggers autoimmune arthritis [27]. The number of CD4^+^ T cells and the Th17/regulatory T (Th17/Treg) ratio in the spleen were higher in the Pg/LA group than in the other groups (data not shown). Therefore, increases in C5a levels by *P. gingivalis* infection may be involved in the exacerbation of arthritis by driving Th17 development.

Clinical findings suggest that ACPA plays an important role in the pathogenesis of RA and correlates with the severity of bone damage in patients with RA [48, 49]. ACPA activates the human complement system via classic and alternative pathways, leading to the release of C5a [50]. Previous studies indicated that elevations in antibodies to *P. gingivalis* are associated with serum levels of ACPA in patients with RA [51]. In the present study, a positive correlation was observed between antibody titers to *P. gingivalis* W83 and serum levels of ACPA, and the Pg/LA group showed an increase in ACPA levels in serum (Fig. 1l). Therefore, elevated C5a levels may have been caused by the ACPA, which was generated by PgPAD. In order to investigate this possibility, the construction of a PAD-deficient strain of *P. gingivalis* is needed. It is true that PgPAD is involved in the generation of ACPA. A previous report indicated that a PAD-deficient strain of *P. gingivalis* reduced the level of ACPA in CIA model mice [52]. However, not only the PgPAD but also other pathogenic factors produced by Pg, including gingipain, fimbriae, cupsule, and LPS, are possible ACPA-inducing factors. For these reasons, there is a limitation to clarify the involvement of PgPAD by using *P. gingivalis* knockout mutants in the progression of RA.

Previous studies indicated that, besides PD and RA, C5a is related to various diseases, including systemic lupus erythematosus, asthma and allergy, atherosclerosis, Alzheimer’s disease, and glomerulonephritis [53]. PD is a highly prevalent chronic inflammatory disease, and many patients with PD are infected with *P. gingivalis* [54]. PD with *P. gingivalis* infection might affect the exacerbation of these diseases via C5a.

## Conclusions

In summary, we demonstrated that oral infection with *P. gingivalis* exacerbated arthritis in the SKG model. Elevations of C5a levels in serum were confirmed in patients with RA and experimental arthritis mice. Elevated C5a levels by an infection with *P. gingivalis* may be partially involved in arthritis via bone destruction by the promotion of osteoclast differentiation. These results suggest that C5a is a target for elucidating the relationship between PD and RA. Further studies are needed in order to clarify the mechanisms by which infection with *P. gingivalis* increases C5a levels in serum as well as the effects of the control of C5a by the treatment with PD on RA.

## Abbreviations

ABL: Alveolar bone loss
ACPA: Anti-citrullinated protein antibody
AS: Arthritis score
BMC: Bone marrow mononuclear cell
C5aRA: C5aR antagonist
CIA: Collagen-induced arthritis
Ctrl: Control
ELISA: Enzyme-linked immunosorbent assay
FBS: Fetal bovine serum
HE: Hematoxylin and eosin
HRP: Horseradish peroxidase
IL: Interleukin
I.p.: Intraperitoneal
LA: Laminarin
LPS: Lipopolysaccharide
M-CSF: Macrophage colony-stimulating factor
Micro-CT: Micro-computed tomography
MMP: Matrix metalloproteinase
NFATc1: Nuclear factor of activated T cells cytoplasmic 1
PAD: Peptidyl-arginine deiminase
PBS: Phosphate-buffered saline
PBST: Phosphate-buffered saline supplemented with 0.05% Tween 20
PD: Periodontal disease
Pg: *Porphyromonas gingivalis*
PgPAD: *Porphyromonas gingivalis* peptidyl-arginine deiminase
RA: Rheumatoid arthritis
RT: Room temperature
RT-PCR: Reverse transcription-polymerase chain reaction
sRANKL: Soluble recombinant receptor activation of nuclear factor kappa-B ligand
Th17: T helper 17
TNF: Tumor necrosis factor
TRAF6: Tumor necrosis factor receptor-associated factor 6
TRAP: Tartrate-resistant acid phosphatase
